# Clinical, histopathologic, subtype, and immunohistochemical analysis of jaw phosphaturic mesenchymal tumors

**DOI:** 10.1097/MD.0000000000019090

**Published:** 2020-02-14

**Authors:** Dongmei Li, Ran Zhu, Lian Zhou, Dingrong Zhong

**Affiliations:** a Department of Pathology, Peking Union Medical College Hospital, Chinese Academy of Medical Sciences & Peking Union Medical College,; b Department of Pathology, China-Japan Friendship Hospital,; c Department of Stomatology, Peking Union Medical College Hospital, Chinese Academy of Medical Science, Beijing, China.

**Keywords:** clinicopathological characteristics, immunohistochemistry, phosphaturic mesenchymal tumors, phosphaturic mesenchymal tumors of mixed connective tissue type, phosphaturic mesenchymal tumors of mixed epithelial and connective tissue type

## Abstract

Jaw phosphaturic mesenchymal tumors (PMTs) are a rare neoplasm with uncertain histogenesis. This study aimed to clarify the clinical and pathological features of jaw PMTs.

We reviewed the clinical records of 39 patients diagnosed with PMTs in the jaws, and investigated clinical and morphologic characteristics, histologic subtypes, and immunophenotypes of all cases.

Microscopic analyses revealed 2 major histologic tumor subtypes: “phosphaturic mesenchymal tumors of mixed epithelial and connective tissue” (PMTMECT), and “phosphaturic mesenchymal tumors of mixed connective tissue” (PMTMCT). PMTMECTs and PMTMCTs accounted for 29 and 10 cases of PMTs, respectively. Most PMTMECT diagnoses were made predominantly in males aged <45 years, and the incidence was similar in both the mandible and maxilla. In contrast, patients with PMTMCTs are predominantly females aged ≥45 years, and all tumors were in the mandible. Histologically, PMTMECT had lower cellularity and a more elongated and spindled mesenchymal component with less elaborate intrinsic microvasculature than PMTMCT. Immunohistochemically, the epithelia of all PMTMECTs was immunoreactive for AE1/AE3. Other immunohistochemical staining of PMTMECTs revealed positive expression of vimentin, SATB2, ERG, CD99, Bcl-2, CD56, S-100, D2-40, CD68, SMA, and CD34 in either one or both components. Immunohistochemical staining of PMTMCTs was diffusely positive for vimentin and a varied ratio of positivity for SATB2, ERG, CD99, Bcl-2, CD56, S-100, D2-40, CD68, SMA, and CD34, but negative for AE1/AE3. Most patients were cured by complete resection, except 2 patients who had repeated recurrences, one of which also had multiple metastasis.

Jaw PMT can be divided into 2 major histological subtypes. PMTMECTs are more common than are PMTMCTs, and can transform into malignant PMTMCTs during the progression. PMTMECTs were more commonly observed in males and the incidence was similar in both the maxilla and mandible. PMTMCTs were almost always observed in the mandible of females. Compared with PMTMCTs, PMTMECTs have an admixture of epithelial components with less prominent vasculature and lower cellularity. There were no statistically significant differences in the expression of immunohistochemical markers except AE1/AE3 between PMTMECTs and PMTMCTs. However, immunohistochemical markers have great significance for differentiating other mesenchymal tumors.

## 1 Introduction

Tumor-induced osteomalacia (TIO), a rare paraneoplastic syndrome, is caused by fibroblast growth factor 23 (FGF23)-secreting mesenchymal tumors.^[1]^ FGF23 overproduction inhibits Na-P cotransporters in the renal proximal convoluted tubule, impairing phosphate reabsorption and leading to phosphate diuresis. Meanwhile, FGF23 inhibits 1-α-hydroxylase activities, reducing renal 1,25-dihydroxy vitamin D production. These events stimulate the release of phosphate and calcium from the bone into the bloodstream as a compensatory mechanism, resulting in systemic bone demineralization. Patients with TIO typically present with bone pain, multiple fractures, and progressive muscular weakness.^[2,3]^ Osteomalacia-associated mesenchymal tumors usually grow slowly and their small size makes them very difficult to detect.^[4]^ Curative surgical resection remains the preferred treatment.^[5,6]^

While TIO can result from different kinds of mesenchymal tumors,^[7]^ phosphaturic mesenchymal tumors (PMTs) are the main cause of TIO.^[1]^ PMTs are typically diagnosed in adults aged between 40 and 50 years,^[8,9]^ and we have previously shown a slight predominance in men (male:female ratio = 1.2:1).^[10]^ PMTs are histologically diverse and frequently infiltrate the capsule, diffusely surrounding soft tissue and/or trabeculae. The tumor cells are spindle or stellate and are usually arranged in a whorled or storiform pattern without or with mild cellular atypia. Adipose cells, myxoid cells, scattered multinucleated giant cells, or cartilage-like cells may also be present. In typical PMT, the nuclei are small round to oval and the nucleoli are inconspicuous with no or minimal nuclear pleomorphism. The tumor is of prominent vascularity and in some cases, focal chondromyxoid or osteoid matrix, “grungy” calcification and areas of erythrocyte extravasation are observed.^[8–10]^

PMTs most commonly involve the extremities followed by the head and neck. Qari et al analyzed 2 cases of head and neck PMTs and concluded, after a comprehensive review of 53 cases in the literature, that the sinonasal cavity represented the most common site, followed by the mandible.^[11]^ Our previous retrospective analysis of 222 PMTs showed that the head and neck was the second most common site, accounting for 32% of all PMTs.^[10]^ Among head and neck PMTs, the jaw area (including the mandible (9%) and the maxilla (5%)) is the most common site (14%), followed by the sinonasal area (13%). Most interestingly, 22 cases, with tumors in the jaw, exhibited mixed mesenchymal and epithelial elements histologically, and the term “phosphaturic mesenchymal tumor, mixed epithelial, and connective tissue” (PMTMECT) type has been proposed.^[10]^

In this study, we collected all PMTs (including the 22 previously reported cases) that were diagnosed at our hospital and located in the jaws and identified 2 major histologic subtypes: PMTMECT, and the typical mixed connective tissue PMTs (PMTMCT). We also compared the differences between the 2 subtypes including clinicopathological characteristics and immunohistochemical profiles, and reviewed the related literature.

## 2 Patients and methods

### 2.1 Patients

We identified 289 cases of PMTs from archived surgical specimens from Peking Union Medical College Hospital (PUMCH), Beijing, China. The diagnosis of “PMTMECT” and “PMTMCT” was established following the criteria established in our previous landmark study^[10]^ and the 2013 World Health Organization classification of soft tissue and bone tumors.^[12]^ Histological and immunohistochemical sections were reviewed independently by 2 experienced pathologists (RZ and DZ). Thirty-nine cases of PMTs, including 22 previously published cases,^[10]^ were located in the jaws. Of these cases, 29 were classified as PMTMECT and 10 as PMTMCT. The study protocol was approved by the PUMCH ethics committee (S-K 762).

### 2.2 Clinical information review

We reviewed the clinical and laboratory records of all patients diagnosed with jaw PMTs and who underwent surgery at PUMCH between 2004 and 2019. We collected information about age, sex, tumor location, tumor size, duration of osteomalacia before biopsy, radiological findings, date of surgery, surgical procedures, time to normophosphatemia after tumor resection, primary diagnosis, and follow-up data.

### 2.3 Immunohistochemistry

Serial sections (5 μm thick) were cut from representative formalin-fixed, paraffin-embedded tumor tissue blocks. After deparaffinization, the sections were subjected to a panel of markers with antibodies against the following markers: somatostatin receptor 2A (SSTR2A) (UMB1, 1:50 dilution; Abcam, Cambridge, UK), FGF23 (polyclonal, 1:2000 dilution; Abcam), SATB2 (EPNCIR130A, 1:50 dilution; Abcam), ERG (EPR3864, 1:1000 dilution; Abcam), CD56 (1B6, prediluted; Leica Biosystems, New Castle, UK), Bcl-2 (Bcl-2/100/D5, 1:50 dilution; Leica), S100 (polyclonal, prediluted; Leica), synaptophysin (27G12, prediluted; Leica), AE1/AE3 (AE1/AE3, prediluted; Leica), vimentin (V9, 1:50 dilution; Dako, Glostrup, Denmark), NSE (BBS/NC/VI-H14, prediluted; Dako), D2-40 (D2-40, 1:50 dilution; Dako), CD99 (12E7, prediluted; Dako), SMA (1A4, 1:50 dilution; Dako), CD34 (QBEnd/10, 1:50 dilution; Dako), CD68 (PG-M1, prediluted; ZsBio, Beijing, China), and Ki-67 (UMAB107, prediluted; ZsBio). Immunohistochemical staining was accomplished using Dako Link 48 autostainer (DAKO) following the manufacturer's instructions. Positive immunoreactivity was nuclear for S100, Ki-67, SATB2, and ERG and cytoplasmic for all other proteins. The tissue sections were scored as negative (<5% positive tumor cells), focally positive (5%–49% positive tumor cells), or diffusely positive (≥50% positive tumor cells). The Ki-67 proliferation index was recorded as the percentage of tumor cells with Ki-67-positive nuclear immunostaining.

## 3 Results

### 3.1 Clinical characteristics of the study population

**Figure 1:**
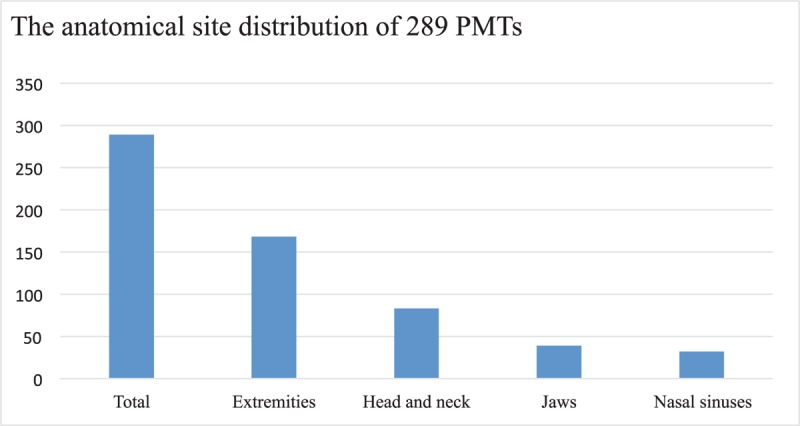
The anatomical site distribution of 289 PMTs.

Fifty eight percent of 289 cases of PMTs were located in the extremities, while the head and neck accounted for 29% of all PMT cases. Among head and neck PMTs, the jaws (13.5%) were the most common location of PMTs, and included the mandible (9%) and the maxilla (4.5%). The second most common head and neck location of PMTs was the nasal sinuses (11%) (Fig. 1). Our pathological review identified 39 jaw PMTs involving the mandible (26 cases) and maxilla (13 cases). All cases were due to TIO and presented with progressive bone pain and muscle weakness with hypophosphatemia, phosphaturia, and abnormal 1, 25-dihydroxy vitamin D. Some cases also showed activity limitation, multiple fractures, tooth loss, and gomphiasis. All tumors were identified by oral physical examination before operation. Jaw PMTs were categorized into 2 morphologic subgroups: PMTMECT (Table 1) and PMTMCT (Table 2).

**Table 1:**
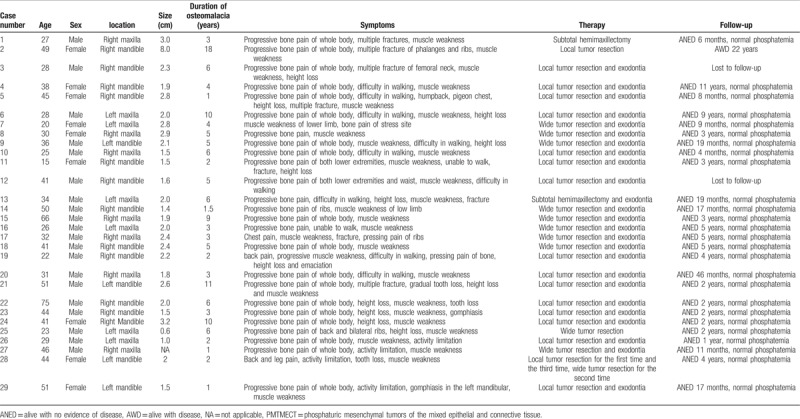
The clinical characteristics and follow-up information of 29 cases of PMTMECT.

**Table 2:**
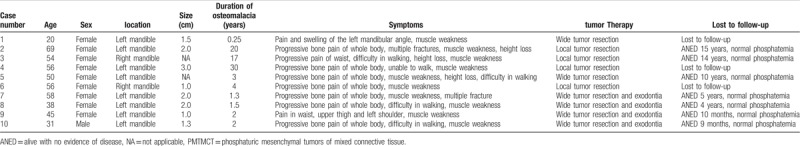
The clinical characteristics and follow-up information of 10 cases of PMTMCT.

Patients with PMTMECT included 20 males and nine females (male: female = 2.2:1). Thirteen of the PMTMECT lesions originated in the maxilla and 16 in the mandible. Most patients were diagnosed at ages less than 45 years (Table 3). Nuclear imaging was performed in 26 of the 29 cases. The tumors were successfully detected by octreotide scanning in 14 cases, whereas ^68^Ga-DOTA-TATE-PET/CT revealed negative or false-positive octreotide scans in the other 12 cases. Twenty-seven (93%) patients underwent (wide) local tumor resection, 2 (7%) patients received subtotal hemimaxillectomy, and 25 (86%) patients underwent exodontia (Table 1). With the exception of 1 patient (patient No. 2), phosphatemia returned to normal in all patients within 9 days of their operation (Table 1).

**Table 3:**
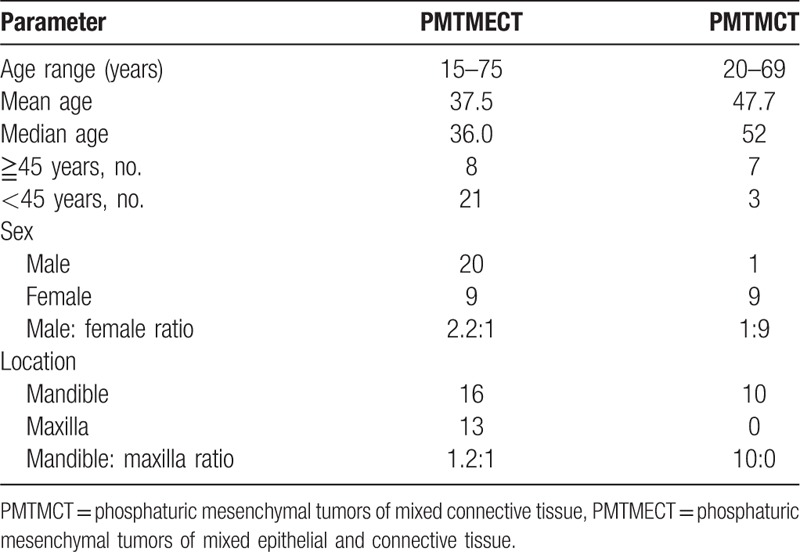
Summary of PMTMECT and PMTMCT clinical differences.

**Figure 2:**
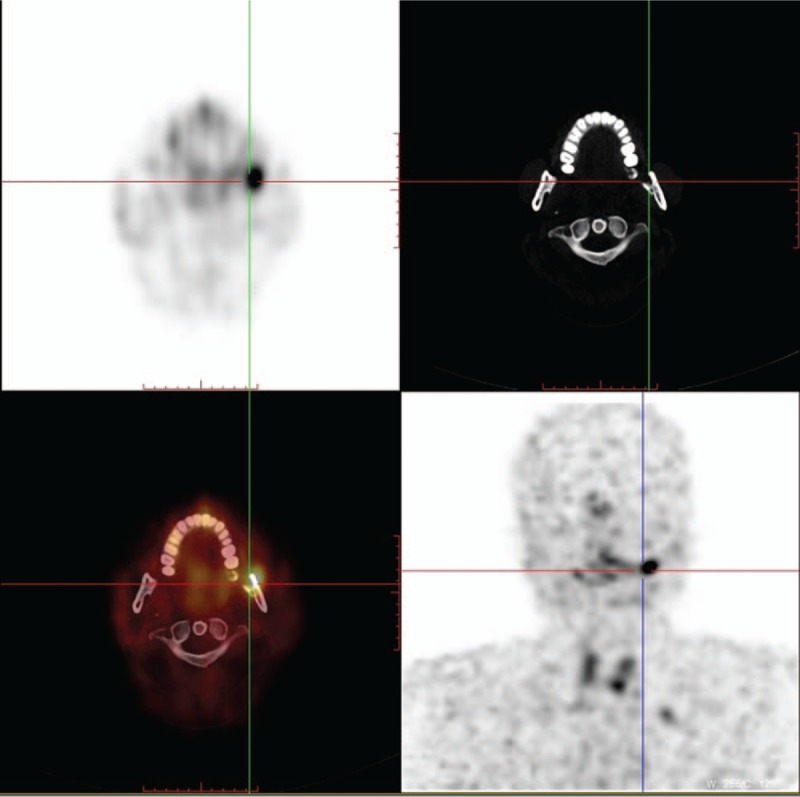
Octreotide scanning was performed in a 31-year-old man who presented with osteomalacia for 2 years. The maximum intensity projection (MIP) image (lower left) reveals a small focus of high somatostatin receptor expression in the left mandible.

Patients with PMTMCT included one male and nine females (male:female = 1:9) and all of the lesions originated in the mandible (Table 2). Most patients with PMTMCT were diagnosed at ages ≥ 45 years (Table 3). Nuclear imaging was performed in 8 of 10 cases. Tumors were successfully detected by octreotide scanning in 6 cases (Fig. 2). All patients received (wide) local tumor resection and 4 (40%) patients underwent exodontia (Table 2). Phosphatemia returned to normal in 8 cases within 1 week of their operation.

Before biopsy, osteomalacia was present in all cases for 1 to 18 years and 3 months to 30 years in patients with PMTMECT and PMTMCT, respectively (Tables 1 and 2).

### 3.2 Histopathologic characteristics

**Figure 3:**
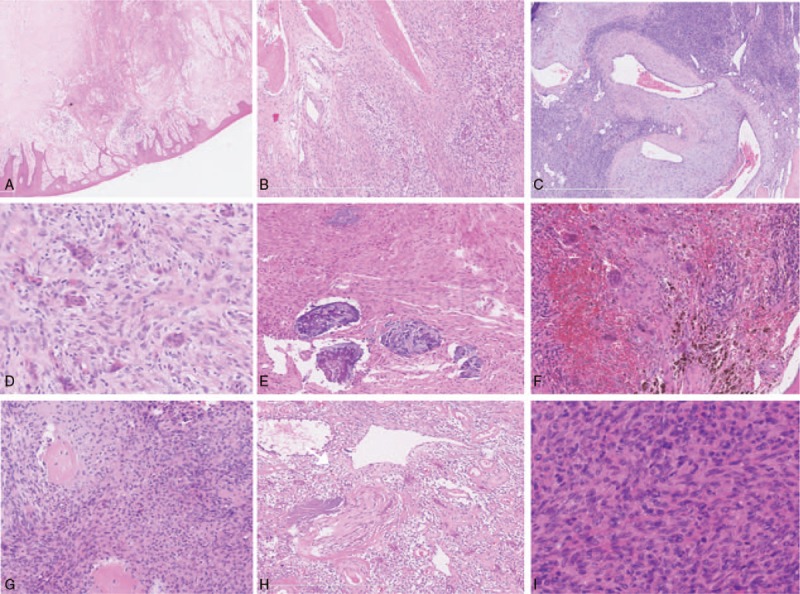
A. The tumor destroys the trabecular meshwork and infiltrates into the surrounding soft tissue and oral mucosa focally (under low magnification). B. PMTMECT contains an admixture of neoplastic epithelial and mesenchymal elements with less prominent vasculature and lower cellularity compared with typical PMTMCT. The mesenchymal component exhibits a more elongated and spindled morphology. The epithelial component of PMTMECT is composed of haphazard and diffuse small, irregular nests throughout the tumor which morphologically resemble odontogenic epithelial nests. Focal osteoid matrix and abnormal thick-walled vessels are present. C. PMTMCT is composed of round to oval, or stellate to spindle, primitive mesenchymal cells. Abnormal thick-walled vessels are readily visible. D. The cytoplasm of PMTMECT is eosinophilic or clear and the nuclei are evenly distributed and unpolarized in neoplastic epithelial cells. E. “Grungy” calcification of PMTMECT. F. Osteoclast-like giant cells are seen in PMTMECT with focal areas of hemorrhages. G. PMTMCT focal osteoid matrix. H Perivascular myxoid changes and “grungy” calcification of PMTMCT. I. Normochromatic nuclei and inconspicuous nucleoli in PMTMCT tumor cells.

Under low magnification (2.5X), the tumors can be seen to disrupt the trabecular meshwork and focally infiltrate the surrounding soft tissue and oral mucosa in all 39 jaw PMTs (Fig. 3A). At higher magnification (10X), the tumors were grouped into 2 major histologic subtypes: PMTMECT and PMTMCT. Histologically, 29 cases were classified as PMTMECT with a mixture of neoplastic epithelial and mesenchymal elements (Fig. 3B), while 10 cases were classified as PMTMCT with round to oval or stellate to spindle primitive mesenchymal cells and small round to oval nuclei (Fig. 3C).

Compared with PMTMCT, PMTMECT had lower cellularity and more elongated and spindled mesenchymal component morphology (Fig. 3B). The epithelial component of PMTMECT haphazardly formed small, irregular nests diffused throughout the tumor morphologically (Fig. 3B). The cytoplasm was eosinophilic or clear and the nuclei were evenly distributed and unpolarized in neoplastic epithelial cells (Fig. 3D). However, in 2 relapsed PMTMECT cases (patient No. 2 and patient No. 28, Table 1), the quantity of epithelial nests declined gradually in serial surgical specimens with higher cellularity and elaborate intrinsic microvasculature; moreover, the tumor mesenchymal elements became less spindle-shaped, and more closely resembled those observed in typical PMTMCT, and the epithelial nests of patient No. 2 disappeared in 2011. Focal osteoid matrix was detected in 86.2% of the patients (25/29) (Fig. 3B), 48.3% (14/29) of the cases showed “grungy” calcification (Fig. 3E), and osteoclast-like giant cells were found focally in areas of hemorrhage in 25% (7/28) of PMTMECT cases (Fig. 3F). Meanwhile, myxoid matrix, perivascular myxoid change, and slate-grey crystals were only observed in 3, 2, and 1 case, respectively. Abnormal thick-walled vessels were detected in 11 cases (Fig. 2B). Dilated thin-walled vessels were observed at the lesion periphery and in the space between the trabecular bone adjacent to the lesion in 12 cases.

Compared with PMTMECT, focal osteoid matrix (Fig. 3G) and perivascular myxoid changes (Fig. 3H) were detected in all ten PMTMCT cases. Abnormal thick-walled vessels (Fig. 3C) and “grungy” calcification (Fig. 3H), were present in nine cases. Myxoid matrix and osteoclast-like giant cells were observed in 5 and 3 cases, respectively.

Cytologically, regardless of PMTMECT or PMTMCT classification, most tumor cells were bland with normochromatic nuclei and inconspicuous nucleoli (Fig. 3D and I). Mitotic figures were absent or rare (0 or 1 in 10 high-powered fields) in 21 PMTMECT cases, and 3 to 5 in 10 high-powered fields in the other 8 cases. However, focal areas with nuclear increasing atypia and high mitotic activity were observed in relapsed PMTMECT cases (patient No. 2 and patient No. 28, Table 1), and the mitotic activity reached to >20 per 10 high-powered fields in patient No. 2. Mitotic figures were absent or rare in 7 cases of PMTMCT (0 or 1 in 10 high-powered fields), and 2 to 6 in 10 high-powered fields in the other three cases. Necrosis was absent in all cases (Table 4).

**Table 4:**
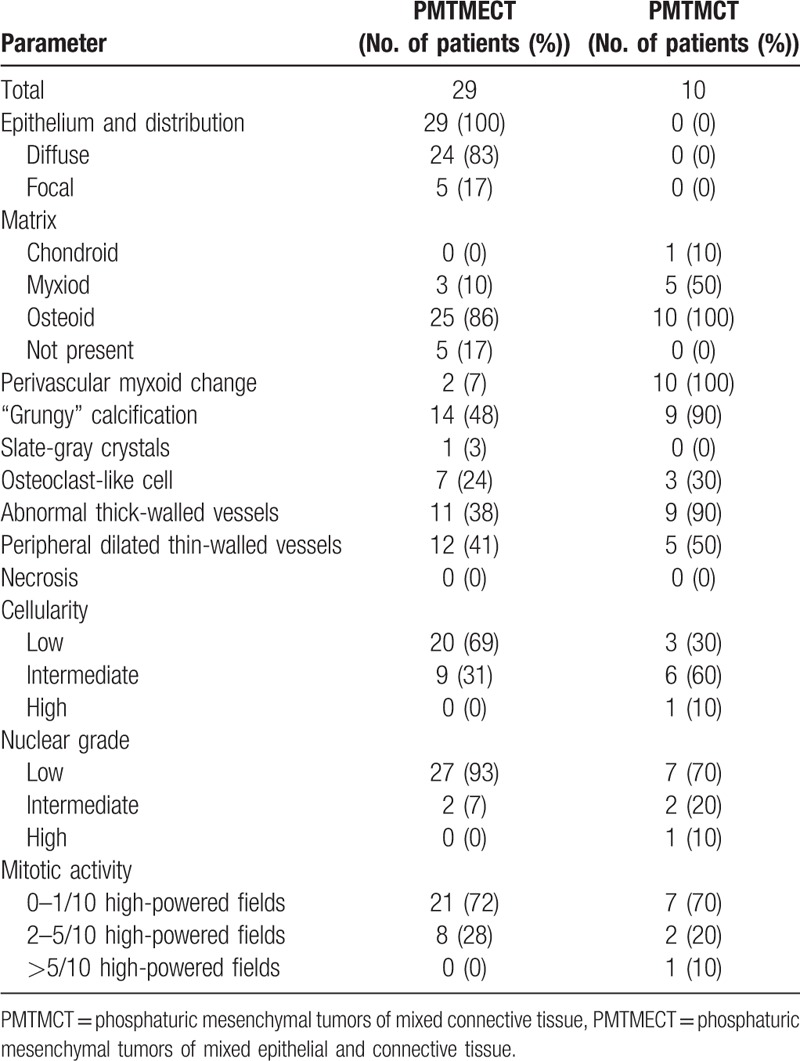
Comparison of PMTMECT and PMTMCT histopathological characteristics.

### 3.3 Immunohistochemical findings

**Figure 4:**
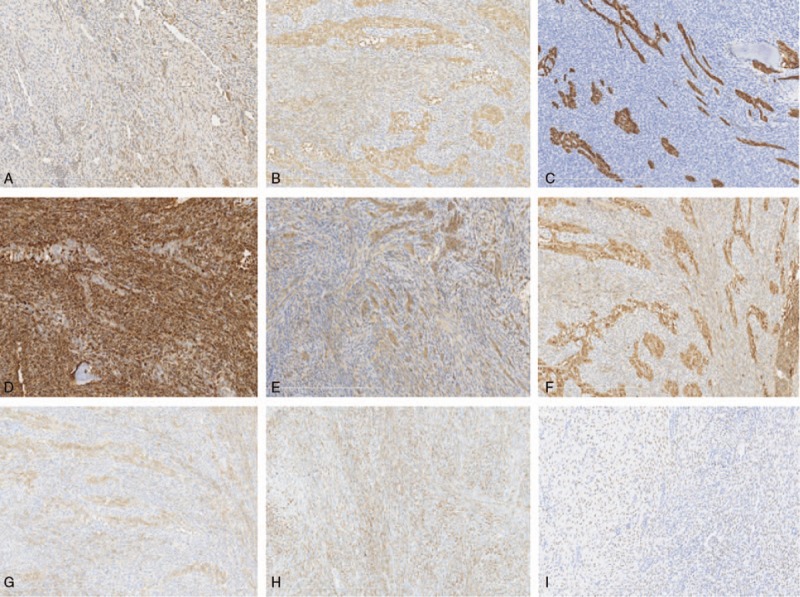
All components are positive for FGF23 (A) and NSE (B). The epithelial and mesenchymal components of PMTMECT show strong diffuse immunoreactivity for AE1/AE3 (C) and vimentin (D), respectively. Most cases are variably positive for CD99 (E), Bcl-2 (F) and CD56 (G) in both components, and the epithelial component exhibits stronger and more diffuse immunoreactivity for FGF23, NSE, CD99, Bcl-2, and CD56 than do the paired connective tissue components. Diffuse or variable focal positive staining for CD68 (H) and SATB2 (I).

The immunohistochemical results of 29 cases of PMTMECT and 10 cases of PMTMCT are summarized in Table 5. Immunohistochemically, all cases were positive for FGF23 (Fig. 4A), SSTR2A, and NSE (Fig. 4B) and negative for desmin, and synaptophysin.

**Table 5:**
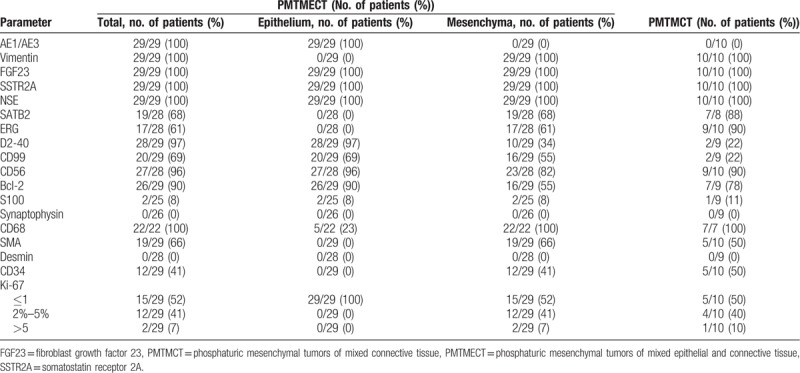
Comparison of PMTMECT and PMTMCT immunohistochemical results.

The epithelial components of all PMTMECT cases showed strong diffuse immunoreactivity for AE1/AE3 and the mesenchymal component was diffusely positive for vimentin (Fig. 4C and D). The PMTMECT tissue specimens were variably positive in either 1 or both components for CD99 (69 and 55% for epithelial and mesenchymal components, respectively) (Fig. 4E), Bcl-2 (90 and 55% for epithelial and mesenchymal components, respectively) (Fig. 4F), CD56 (96 and 82% for epithelial and mesenchymal components, respectively) (Fig. 4G), and D2-40 (97 and 34% for epithelial and mesenchymal components, respectively). S100 was positive in both epithelial and mesenchymal components in 2 cases. Immunoreactivity of the epithelial components was typically stronger and more diffuse than the immunoreactivity of paired connective tissue components for FGF23, NSE, CD99, Bcl-2, and CD56 (Fig. 4A, B, and E–G), D2-40, and S100. A diffuse or variable focal positive staining was observed only in the connective tissue components of PMTMECT cases for D68 (22/22, 100%) (Fig. 4H), SATB2 (19/28, 68%) (Fig. 4I), ERG (17/28, 61%), SMA (19/29, 66%), and CD34 (12/29, 41%).

**Figure 5:**
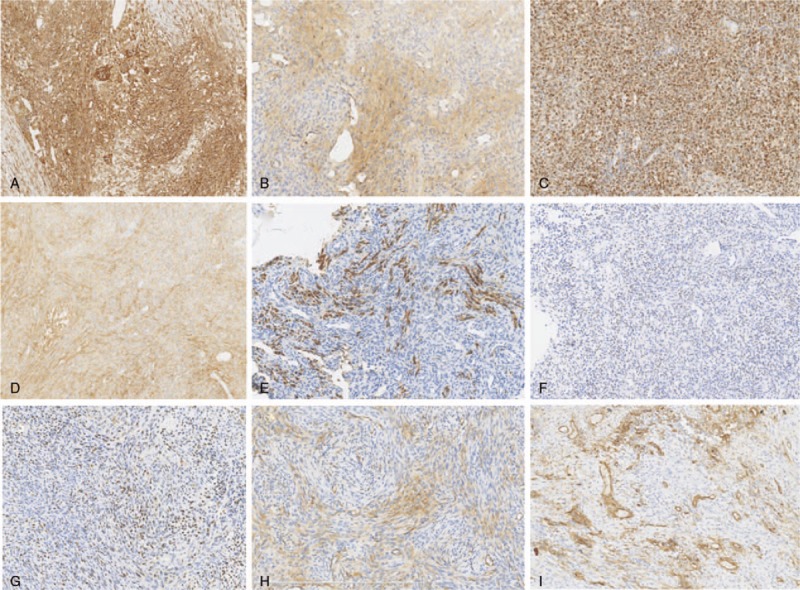
PMTMCT is variably, but diffusely, positive for vimentin (A), CD99 (B), Bcl-2 (C), CD56 (D), and S-100 (E), and focally positive SATB2 (F), ERG (G), SMA (H), and CD34 (I).

PMTMCT samples were also variably diffusely positive for vimentin (10/10, 100%) (Fig. 5A), CD99 (2/9, 22%) (Fig. 5B), Bcl-2 (7/9, 78%) (Fig. 5C), CD56 (9/10, 90%) (Fig. 5D), S-100 (1/9, 11%) (Fig. 5E), and CD68 (7/7, 100%) and variably focally positive for SATB2 (7/8, 88%) (Fig. 5F), ERG (9/10, 90%) (Fig. 5G), SMA (5/10, 50%) (Fig. 5H), and CD34 (5/10, 50%) (Fig. 5I). All PMTMCT cases were negative for AE1/AE3 (0/10).

The Ki-67 proliferation index of the primary tumors ranged from <1 to 10% and <1 to 20% in PMTMECTs and PMTMCTs, respectively. The Ki-67 labelling index increased from ≤1 to 25% and from ≤1 to 15% during tumor progression in patient No. 2 (Table 1) and patient No. 28 (Table 1), respectively.

### 3.4 Follow-up information

Twenty-seven PMTMECT cases were followed up from 4 months to 22 years. Twenty-five (25/27, 92.6%) patients survived with normal phosphatemia, and no local recurrence or distant metastasis were reported. Two patients (2/27, 7.4%) had repeated recurrences and one of them had multiple metastasis at the last follow-up (Table 1). Although patient No. 2 (Table 1) had undergone seven operations for local tumor resection and nuclear imaging suggested multiple metastases, he was still alive at the last follow-up (June 2019). Patient No. 28 had multiple recurrences and received 2 operations for local tumor resection and one operation for wide tumor resection since 2015, and was recovering well with normal phosphatemia at the last follow-up (June 2019).

Seven cases of PMTMCT were followed up at various times ranging from 9 months to 14 years. All seven patients survived with normal phosphatemia. No local recurrence or distant metastasis were reported at the last follow-up (Table 2).

## 4 Discussion

PMT was first reported in 1947,^[13]^ but its association with osteomalacia was only recognized in 1959.^[14]^ Evans et al^[15]^ and Olefsky et al^[16]^ later demonstrated distinctive TIO lesions that differentiate PMT from other soft tissue and bone neoplasms.

PMTs occur in adults with equal gender distribution and most commonly involve the extremities, followed by the head and neck. According to previous literature, in the head and neck, the sinonasal cavity was the most common location for PMTs, followed by the mandible.^[11,17]^ Our previous results also show that the sites most affected by PMTs are the extremities, followed by the head and neck, that the median age of those affected is 44 years, and that there is a slight predominance in men.^[10]^ For head and neck PMTs, our study revealed that the jaw (13%) was the are most often affected area (including the mandible (9%) and the maxilla (4%)), followed by the nasal sinuses (11%).

In 1987, Weidner and Santa Cruz^[18]^ categorized PMT into 4 subtypes: PMTMCT, osteoblastoma-like, ossifying fibroma-like, and non-ossifying fibroma-like types.^[18,19]^ Subsequently, we proposed a new variant of PMT, PMTMECT, that was only located in the jaws.^[10]^ In this study, we found 39 cases of PMT located in the jaws and involving the mandible (26 cases) and maxilla (13 cases). Twenty-nine of the cases (74%) were classified as PMTMECT while the other 10 cases (26%) were classified as PMTMCT. We compared jaw PMTMECT and PMTMCT and found both similar and different clinicopathologic features.

Clinically, all cases of PMT in the jaws were characterized with TIO and hypophosphatemia, and presented with progressive bone pain, muscle weakness, and abnormal 1, 25-dihydroxy vitamin D. Therefore, the clinical presentation of the lesion is important and could assist diagnosis at the time of biopsy assessment. PMTs in other sites are generally small, deeply located, and may be missed by routine clinical examination.^[17]^ In our study, all PMT cases involving the jaws were detected by oral examination or were first discovered by patients themselves due to the superficial tumor locations. Most patients diagnosed as having PMTMECT were predominantly male, aged <45 years, and the incidence in the maxilla and mandible were similar. In contrast, PMTMCTs were predominant in female patients, ≥45 years, and all tumors were in the mandible.

Histologically, the tumors of all cases disrupted the trabecular meshwork and infiltrated the surrounding soft tissue and oral mucosa focally. PMTMCT located in the jaws is only composed of mesenchymal components, while PMTMECT is characterized by a mixed proliferation of epithelial nests arranged haphazardly among the mesenchymal components. Moreover, the mesenchymal components of PMTMECT has less prominent vasculature and lower cellularity, while PMTMCT typically consists of primitive mesenchymal cells with a large number of small capillaries and higher cellularity. Interestingly, we observed that the quantity of epithelial nests declined gradually in serial surgical specimens from 2 relapsed PMTMECT cases (patient No. 2 and patient No. 28, Table 1) with more capillaries and higher cellularity. The mesenchymal components of relapsed PMTMECTs became less spindle-shaped, and more closely resembled those observed in typical PMTMCT and the nuclei became increasingly atypical, meanwhile, the mitotic activity and the Ki-67 labelling index gradually increased; the epithelial nests of patient No. 2 finally disappeared in 2011. Focal osteoid matrix is found in most cases of PMTMECTs, but calcification and osteoclast-like giant cells are not common. Moreover, myxoid matrix and perivascular myxoid changes are rare. Other features of PMTMCTs, including intralesional fat and microcystic changes, were not observed in PMTMECTs in this study.

In all cases, tumors showed diffuse positive immunohistochemical staining for FGF23, SSTR2A, and NSE. Although FGF23, SSTR2A, and NSE are not specific for PMTs,^[20–22]^ the combination of these immunophenotypes (FGF23+, SSTR2A+, and NSE+) rules out other tumors, including solitary fibrous tumor/hemangiopericytoma, synovial sarcomas, and schwannomas. Negative staining for these three markers (FGF23-, SSTR2A-, and NSE-) could be used to rule out phosphaturic mesenchymal tumors.^[20–22]^ Compared with PMTMCTs, some PMTMECT tumor cell nests were positive for AE1/AE3 and negative for vimentin, indicating an epithelial component presence. Mesenchymal PMTMECT cells and all PMTMCT cells are diffusely positive for vimentin and negative for AE1/AE3. Similar to our previous report,^[10]^ we found that PMTMECTs and PMTMCTs were variably positive for CD99, Bcl-2, CD56, D2–40, and S-100. Among the PMTMECTs, the epithelial component was more strongly and/or more diffusely positive for FGF23, NSE, CD99, Bcl-2, CD56, D2-40, and S-100 than was the paired mesenchymal component, indicating that the PMTMECT epithelial component possesses neoplastic traits and shares an origin with the mesenchymal component. Variable focal positive staining for SATB2, ERG, SMA, and CD34 was observed in the PMTMCT cases, but was only partially evident in the connective tissue components of PMTMECT cases. Immunohistochemistry of SATB2, a marker of osteoblastic and chondroblastic differentiation,^[23–25]^ revealed the inherent tendency of PMTMCT cells and the mesenchymal component of PMTMECT cells for osteoblastic differentiation. ERG and CD34 positivity showed vascular differentiation of PMTs. There were more ERG and CD34 positive PMTMCT cells than there were ERG and CD34 positive mesenchymal PMTMECT cells, potentially explaining the why PMTMECT has less prominent vasculature. PMTMCTs and PMTMECTs located in the jaws are positive for the SSTR2A, NSE, and CD56 neuroendocrine markers, indicating that neuroendocrine cell differentiation in these tumors requires further study. Although PMTs have been reported to be negative for CD68, S-100, and CD34 staining,^[11,26,27]^ in this study, and our previous study, the tumor cells of PMTMCTs and PMTMECTs showed consistent diffuse positive staining for CD68 and variable focal positive staining for S-100 and CD34. Taken together, these results show that, in the majority of the cases analyzed, PMTs are characterized by a distinctive and wide immunophenotypic spectrum (vimentin+/FGF23+/SSTR2A+/NSE+/CD99+/D2-40+/Bcl-2+/CD34+/SATB2+/ERG+/CD56+/CD68+), and that positivity of AE1/AE3 could distinguish PMTMECTs from PMTMCTs.

It is very important to differentiate PMTMECTs and PMTMCTs from other histological mimickers (such as ossifying fibromas, osteosarcoma, fibrohistiocytic tumor, myofibroblastic tumor, and solitary fibrous tumor) due to treatment and prognosis differences. The wide immunophenotype (vimentin, SSTR2A, FGF23, NSE, CD99, CD56, Bcl-2, D2-40, AE1/AE3, CD34, CD68, ERG, and SATB2) is very distinctive and valuable for the differential diagnosis. The epithelial nests in PMTMECTs can be misinterpreted as giant cells, leading to the erroneous diagnosis of giant cell tumors or giant cell granulomas,^[11,28,29]^ but AE1/AE3 is helpful in the differential diagnosis of giant cell lesions and PMTMECTs.

We reviewed the literature on jaw PMTs in PubMed from 1972 to 2019. Eighteen cases of osteomalacia-associated mesenchymal tumors of the jaws were reported between 1972 and 2019.^[4,6,11,19,28–40]^ Expectedly, the mandible was most often involved (13/18, 72%), followed by the maxilla (5/18, 28%). Local invasion by jaw PMTMCT and PMTMECT was observed and most patients were cured by complete resection. Fourteen cases were reported in our previous study.^[10]^ Six of these cases most closely fit our proposed definition of PMTMECT with significant male predominance (5:1) and a median age of 42 years.^[4,19,29–32]^ In a relapsed PMTMECT case,^[30]^ there was a reduction in the epithelial component quantity and finally the recurrent and lung-metastasized tumors were composed solely of neoplastic spindle cells. Six other cases may fit the proposed criteria of PMTMECT with a significant male predominance (5:1) and a median age of 33 years.^[11,28,29,33–35]^ Four cases probably fit the proposed definition of PMTMCT with a significant female predominance (female: male = 4:0) and a mean age of 46 years.^[6,36,39,40]^ The tumor occurred in the mandible in 3 cases^[6,36,40]^ and in the maxilla in 1 case.^[39]^ The 4 cases of PMTMCT were followed up from 1 month to 7 years, and all patients survived with normal phosphatemia and no local recurrence or distant metastasis was seen. Two cases had insufficient data for classification.^[37,38]^

In the jaws, all cases of PMTMCT were cured, both in our series and in the literature, while 93% (27/29) of PMTMECT cases were cured in our series and 92% (11/12) were cured in the literature after operation, and the phosphatemia returned to normal. Three cases of PMTMECT (2 cases in our series and one case in the literature^[30]^) had repeated recurrences and 2 of the patients had multiple metastases. Repeated recurrences of PMTMECT could cause malignant transformation and metastasis.^[30]^ A diminution and gradual disappearance of the epithelial component was observed during the aggressive PMTMECT recurrence, progression, and metastasis with nuclei becoming increasingly atypical and mitotic activity increasing. Of the three aggressive PMTMECTs, 66.7% (2/3, 1 case in our series and 1 case in the literature^[30]^) translated into malignant PMTMCTs in the process of malignant transformation. Taken together, despite being locally invasive in most cases, PMT of the jaws is usually benign. Most patients are cured by complete resection, and there is an immediate and dramatic clinical and biochemical improvement after tumor resection. However, aggressive PMTMECT cases should be investigated with caution, as these indicate a malignant transformation with multiple local recurrences or metastases.^[10,30]^

In summary, jaw PMTs are distinctive low-grade tumors exhibiting two major histologic subtypes. In the jaws, PMTMECTs are more common than are PMTMCTs, and aggressive PMTMECTs can transform into malignant PMTMCTs during the progression of the disease. Additionally, PMTMECT is more common in males and the incidence of PMTs is similar in the maxilla and the mandible. In contrast, PMTMCT is more frequent in females and mostly occurs in the mandible. Compared with PMTMCT cases, PMTMECT patients are younger. Moreover, PMTMECT only occurs in the jaws and has an admixture of epithelial components with less prominent vasculature and lower cellularity. So far, AE1/AE3 is the only differentiating immunohistochemical marker between PMTMECT and PMTMCT. With the exception of AE1/AE3, there was no statistically significant difference between the expression of immunohistochemical markers in PMTMECTs and PMTMCTs. However, immunohistochemical markers are of great importance for differentiating other tumor types.

## Acknowledgments

We thank the medical technologists of Department of Pathology, Peking Union Medical College Hospital for vigorous technical assistance.

## Author contributions

**Data curation:** Dongmei Li, Ran Zhu.

**Formal analysis:** Dongmei Li, Ran Zhu, Lian Zhou.

**Investigation:** Dongmei Li, Ran Zhu.

**Methodology:** Dingrong Zhong.

**Supervision:** Dingrong Zhong, Lian Zhou.

**Validation:** Dingrong Zhong.

**Writing – original draft:** Ran Zhu.

**Writing – review & editing:** Dongmei Li, Dingrong Zhong.
